# The variability of motor evoked potential latencies in neurosurgical motor mapping by preoperative navigated transcranial magnetic stimulation

**DOI:** 10.1186/s12868-016-0321-4

**Published:** 2017-01-03

**Authors:** Nico Sollmann, Lucia Bulubas, Noriko Tanigawa, Claus Zimmer, Bernhard Meyer, Sandro M. Krieg

**Affiliations:** 1 Department of Neurosurgery, Klinikum rechts der Isar, Technische Universität München, Ismaninger Str. 22, 81675 Munich, Germany; 2 TUM-Neuroimaging Center, Klinikum rechts der Isar, Technische Universität München, Munich, Germany; 3 Faculty of Linguistics, Philology, and Phonetics, University of Oxford, Walton Street, Oxford, OX1 2HG UK; 4 Section of Neuroradiology, Department of Radiology, Klinikum rechts der Isar, Technische Universität München, Ismaninger Str. 22, 81675 Munich, Germany

**Keywords:** Brain tumor, Cortical mapping, Electromyography, Motor evoked potentials, Navigated transcranial magnetic stimulation, Presurgical motor mapping

## Abstract

**Background:**

Recording of motor evoked potentials (MEPs) is used during navigated transcranial magnetic stimulation (nTMS) motor mapping to locate motor function in the human brain. However, factors potentially underlying MEP latency variability in neurosurgical motor mapping are vastly unknown. In the context of this study, one hundred brain tumor patients underwent preoperative nTMS-based motor mapping of the tumor hemisphere between 2010 and 2013. Fourteen predefined predictor variables were recorded, and MEP latencies of abductor pollicis brevis muscle (APB), abductor digiti minimi muscle (ADM), and flexor carpi radialis muscle (FCR) were analyzed using linear mixed-effect multiple regression analysis with the forward step-wise model comparison approach.

**Results:**

Common factors (relevant to APB, ADM, and FCR) for MEP latency variability were gender, most likely due to body height, and antiepileptic drug (AED) intake. Muscle-specific factors (relevant to APB, ADM, or FCR) for MEP latency variability were resting motor threshold (rMT), tumor side, and tumor location.

**Conclusions:**

Based on a large cohort of neurosurgical patients, this study provides data on a wide range of clinical factors that may underlie MEP latency variability. The factors that significantly contributed to MEP latency variability should be standardly recorded and taken into consideration during neurosurgical motor mapping.

## Background

Transcranial magnetic stimulation (TMS) is a noninvasive tool that can be applied to systematically map the human cortex with the aim of localizing specific function. Thanks to the combination of precise navigation systems and TMS, navigated TMS (nTMS) becomes possible, which can be used for reliable cortical motor mapping in the context of presurgical planning among patients with brain tumors [1–3]. In this context, it has already been shown that nTMS-based motor maps correlate well with intraoperative direct cortical stimulation (DCS) mapping, especially when compared to other common preoperative mapping modalities such as functional magnetic resonance imaging (fMRI) or magnetoencephalography (MEG) [4–7]. Furthermore, recent data provided the first evidence that patients with brain tumors might benefit from nTMS in terms of clinical outcome and survival, thus potentially expanding the initial role of nTMS as a mere preoperative planning tool [8–10].

During neurosurgical nTMS-based motor mapping, the functional motor area is typically identified and spatially enclosed by separating cortical areas that gave rise to motor evoked potentials (MEPs) during stimulation (motor-positive spots) from those for which no adequate responses were detected (motor-negative spots). However, to be able to achieve accurate motor maps that facilitate reliable preoperative planning, improved clinical outcome, and perioperative risk stratification, precise definitions of criteria that allow for distinguishing between motor-positive and motor-negative spots are required. Among others, MEP amplitudes and MEP latencies are most commonly used for this purpose [4–6]. Whereas MEP latencies have principally shown to remain comparatively stable, MEP amplitudes vary considerably from stimulus to stimulus in patients with pathologies of the nervous system as well as in healthy subjects [11–14]. Although MEP latencies are considered more robust and, therefore, presumably even more reliable for separating motor-positive from motor-negative stimulation points, data on normative MEP latency values and factors underlying MEP latency variability in neurosurgical patients have only been provided by one study so far [14]. However, this study primarily revealed negative results in the sense that no factors except gender were identified for MEP latency variability [14]. Furthermore, the overall topic of MEP amplitudes, latency variability, and influencing factors has been primarily addressed by non-navigated TMS studies with only a few exceptions so far [11, 12, 15–17]. However, non-navigated TMS does not allow for precise control of coil angulation, orientation, and localization of the stimulation with respect to individual cortical anatomy. Yet, only slight variations in coil placement can already lead to different responses, which demonstrates the need for updated values of MEP characteristics by the use of nTMS [18]. Taking these aspects into account, the current study addresses the following topics:

1. While there is some literature available on MEP characteristics derived from nTMS among healthy subjects [13, 19], data derived from neurosurgical patients are rare. Thus, the present study aims to provide further evidence on MEP latency distributions among patients with brain tumors.
2. Except for one study, factors that might interfere with MEP characteristics during neurosurgical motor mapping have not been assessed [14]. Thus, the present study examines clinical factors that may underlie MEP variability while expanding the range of factors that have been taken into account previously. Most of these factors are specific to brain tumors and are not examinable in healthy cohorts.

## Methods

### Patients and procedures

The present study was conducted among 100 patients in our neurosurgical department. The same cohort has been investigated with a different purpose in previous studies [20, 21].

According to our study protocol, inclusion criteria included individuals above age 18, with written informed consent, and with brain lesions affecting motor areas according to anatomical magnetic resonance imaging (MRI). Exclusion criteria included individuals below 18 years old, general TMS exclusion criteria (e.g., cochlear implant, pacemaker, deep brain stimulation electrodes), and plegia.

Regarding the experimental setup, the enrolled patients first underwent detailed clinical examinations including assessment of motor strength according to a standardized protocol with respect to the British Medical Research Council (BMRC) scale. Then, cranial MRI followed by nTMS-based motor mapping was conducted. All nTMS mapping sessions were systematically analyzed to be able to provide data on MEP latency distributions and characteristics among neurosurgical patients. Moreover, various patient-related, tumor-related, and mapping-related characteristics were systematically recorded for later regression analysis to identify factors that may underlie MEP latency variability.

### Cranial imaging

Among other clinical sequences, our scanning protocol consisted of a three-dimensional gradient echo sequence (TR/TE: 9/4 ms, 1 mm^3^ isovoxel covering the whole head, 6 min 58 s acquisition time) with and without intravenous contrast administration for navigation purposes during nTMS. Imaging was done on a 3 Tesla scanner by use of an 8-channel phased array head coil (Achieva 3T, Philips Medical Systems, The Netherlands B.V.).

### Motor mapping

Motor mapping by nTMS was performed with the Nexstim eXimia NBS system, version 3.2 or 4.3 (Nexstim Oy, Helsinki, Finland). A biphasic figure-of-eight magnetic coil was used for all mappings, and an integrated infrared tracking system allowed for real-time navigation during stimulation. Motor responses were continuously monitored using the integrated electromyography (EMG) system with six channels in total. All examinations were conducted according to a validated stimulation protocol by experienced investigators [5, 10, 20–22].

During nTMS, the patients sat in an adjustable chair with armrests, and pregelled surface electrodes were placed over the abductor pollicis brevis muscle (APB), abductor digiti minimi muscle (ADM), flexor carpi radialis muscle (FCR), biceps brachii muscle (BCS), tibialis anterior muscle (TA), and gastrocnemius muscle (GCN) contralateral to the brain lesion (Neuroline 720, Ambu, Ballerup, Denmark). EMG recording of each muscle was derived from a pair of electrodes with the first electrode being placed on the respective muscle belly and the second electrode being placed on a nearby bony or tendinous part according to the recommendations of the system’s manufacturer. The second electrode was always placed distal to the first electrode for each muscle, and the inter-electrode distance was in the range of few centimeters.

Then, the resting motor threshold (rMT) as the lowest stimulation intensity that elicits MEPs over 50 µV in amplitude in at least 50% of stimulation trials in a relaxed muscle was determined by motor mapping of the cortical representation of the APB [23]. Subsequent to rMT determination, motor mapping of the hemisphere with the brain tumor was performed. In this context, we chose 110% rMT for mapping of upper extremity (UE) muscles, while the lower extremity (LE) was assessed with at least 130% rMT according to previous reports [5, 10, 20–22]. Mapping was performed with a distance of less than 1 cm between single stimulation points, and the electric field induced by the stimulating nTMS coil was oriented perpendicular to the mapped gyrus for UE mapping. During the whole stimulation procedure, patients were advised to relax, and mapping was only performed when preinnervation levels of all recorded muscles were clearly below 50 µV in amplitude, thus avoiding false-positive stimulation spots.

After each mapping session, post hoc analysis was done as described earlier [4–6, 14]. In this context, only mapping points with MEP amplitudes greater than or equal to 50 µV were taken into account for further analysis, and, consequently, this criterion was used to distinguish between motor-positive (MEP amplitudes ≥50 µV) and motor-negative stimulation points (MEP amplitudes <50 µV). Again, this approach follows the procedures established during earlier investigations on motor mapping in patients with brain tumors [5, 10, 20–22]. Hence, only motor-positive stimulation spots of each patient were further considered during MEP latency analyses of the present study.

### Patient data collection

To identify factors that may underlie MEP latency variability, 14 predefined predictor variables were recorded. These variables were collected from the medical charts or assessed by a standardized questionnaire subsequent to the mappings.

In this context, patient-related parameters contained gender (male = M, female = F), age at exam, antiepileptic drug intake (AEDs: no AED = NA, levetiracetam = L, other specified AEDs = SA, AED status not known = NK, unspecified AEDs = UA), and presence of motor deficits (no deficit = ND, deficit = D). Furthermore, tumor-related factors included tumor location (Rolandic = RO, frontal = FR, parietal = PA, postcentral gyrus = PoG, or temporal = TE), tumor side (left hemisphere = LH, right hemisphere = RH), dominancy of the tumor hemisphere (non-dominant = NDO, dominant = DO), tumor-related edema (no edema = NE, edema = E), tumor entity (astrocytoma WHO grade II = II, astrocytoma WHO grade III = III, astrocytoma WHO grade IV = IV, metastasis = ME, other entities = X), tumor recurrence (no recurrence = NR, recurrence = R), and tumor volume. Additionally, a predefined set of mapping-related parameters was taken into account, which consisted of the rMT intensity, mapped muscles (APB, ADM, FCR), and year of mapping (2010–2013 = Y10, Y11, Y12, Y13). The year of mapping can be regarded as an important external factor related to the skills of the investigators, because lower MEPs might potentially arise when some coil operators were more experienced than others.

### Statistical analysis

All statistical data analysis was performed by using R, version 3.1.1, in combination with the MASS package and the effects package [24, 25] (The R Foundation for Statistical Computing, Vienna, Austria).

For documentation and reporting of basic patient and mapping characteristics, absolute frequencies, means ± standard deviation (SD), and ranges were calculated. Regarding MEP latency data, Shapiro–Wilk normality test was initially performed to assess whether MEP latency data were normally distributed. In case of non-normal data distribution, descriptive statistics were summarized by percentile rank scores (minimum, median, maximum, and quartile scores), and ex-Gaussian measures were calculated and used for further MEP latency analyses. The ex-Gaussian distribution is a mathematical convolution of the normal (Gaussian) and exponential distributions and has three parameters: mu and sigma, representing the mean and SD of the Gaussian distribution, and tau, representing the mean and SD of the exponential distribution. In this context, mu represents the mode of the normally distributed part, whereas sigma is the measure of dispersion in the normally distributed part.

Since previous data among neurosurgical patients have shown gender-dependent significant differences in MEP latencies [14], the initial analyses for MEP distributions were done separately for males and females. Furthermore, to investigate the factors underlying the variability in MEP latency, linear mixed-effect multiple regression analysis with the forward step-wise model comparison approach was performed. To test for statistical significance, a Chi-squared test was applied, and p < 0.05 was defined as the level of significance.

For initial assessment and illustration of MEP latency distributions, line graphs and boxplots were generated. Concerning factors that proved to significantly contribute to MEP latency variability, effect plots including confidence intervals (CIs) were prepared subsequent to linear regression analysis.

## Results

### Patient and mapping characteristics

Overall, 100 patients were enrolled in the present investigation, and nTMS-based motor mapping was achieved successfully in all subjects. During stimulation, no adverse events occurred. Relevant patient details, mapping parameters, and the set of predefined clinical factors are displayed in Table 1. The same patient cohort has been investigated with a different purpose in previous studies [20, 21].

**Table 1:** Patient and mapping characteristics

	Male	Female	All
Number of patients	57	43	100
Age at exam (in years)	54.0 ± 13.9 (20–83)	54.2 ± 15.7 (19–84)	54.1 ± 14.7 (19–84)
AEDs			
NA	26	27	53
L	19	11	30
SA	4	1	5
NK	5	3	8
UA	3	1	4
Motor deficit			
ND	41	31	72
D	16	12	28
Tumor location			
RO	19	16	35
FR	15	9	24
PA	11	8	19
PoG	11	6	17
TE	1	4	5
Tumor side			
LH	23	17	40
RH	34	26	60
Dominancy			
NDO	28	25	53
DO	26	16	42
Edema			
NE	37	31	68
E	20	12	32
Tumor entity			
II	8	6	14
III	4	6	10
IV	21	17	38
ME	14	11	25
X	10	3	13
Tumor recurrence			
NR	44	34	78
R	13	9	22
Tumor volume (in cm^3^)	27.2 ± 5.4 (19.9–42.9)	26.1 ± 5.0 (20.3–39.9)	26.7 ± 5.2 (19.9–42.9)
rMT (in %)	31.9 ± 7.5 (20–59)	36.4 ± 10.6 (22–72)	33.8 ± 9.3 (20–72)
Year of mapping			
Y10	13	16	29
Y11	14	6	20
Y12	10	5	15
Y13	20	16	36

Overview on patient and mapping characteristics by gender, including age at exam, antiepileptics (AEDs: *NA * no AED, *L* levetiracetam, *SA* other specified AEDs, *NK* AED status not known, *UA* unspecified AEDs), and presence of motor deficits (*ND* no deficit, *D* deficit). Furthermore, tumor location (*RO* Rolandic, *FR* frontal, *PA* parietal, *PoG* postcentral gyrus, *TE* temporal), tumor side (*LH* left hemisphere, *RH* right hemisphere), dominancy of the tumor hemisphere (*NDO* non-dominant, *DO* dominant), tumor-related edema (*NE* no edema, *E* edema), tumor entity (*II* astrocytoma WHO grade II, *III* astrocytoma WHO grade III, *IV *astrocytoma WHO grade IV, *ME* metastasis, *X *other entities), tumor recurrence (*NR* no recurrence, *R* recurrence), and tumor volume are displayed. In addition, resting motor threshold (rMT) values and year of mapping (*Y10* exam year 2010, *Y11* exam year 2011, *Y12* exam year 2012, *Y13* exam year 2013) are shown. All values are presented as number of patients, mean ± standard deviation (SD), or ranges. Dominancy data do not add up to 100 patients since it was not assessed in five patients

### MEP latency distribution

**Fig. 1:**
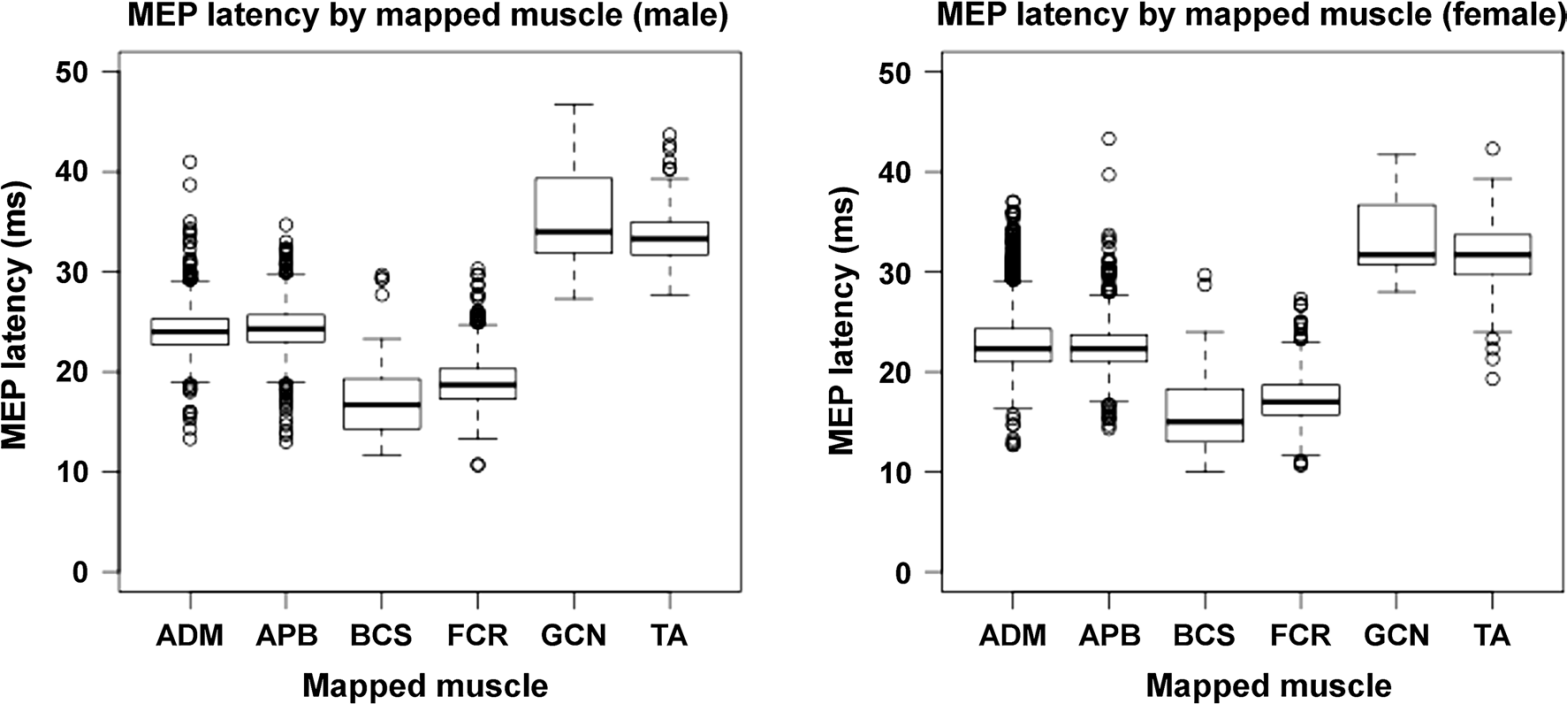
Boxplots showing non-normally distributed motor evoked potential (MEP) latencies for mapped muscles of both genders. In the present study, the abductor pollicis brevis muscle (APB), abductor digiti minimi muscle (ADM), flexor carpi radialis muscle (FCR), biceps brachii muscle (BCS), tibialis anterior muscle (TA), and gastrocnemius muscle (GCN) were mapped

**Fig. 2:**
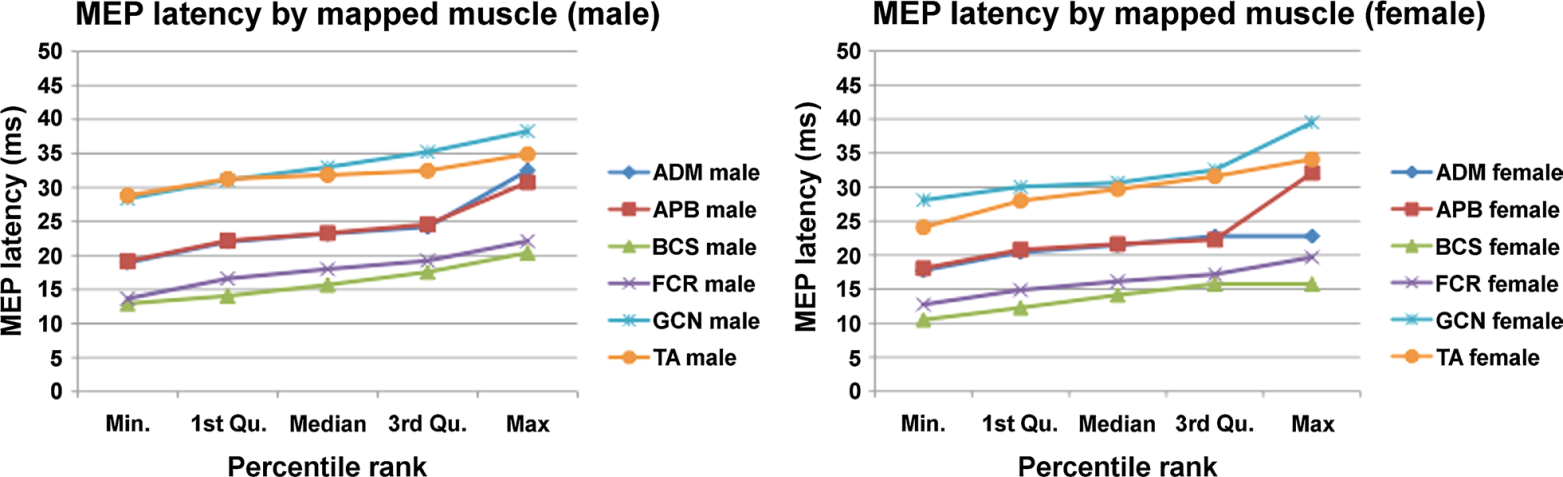
Graphs showing motor evoked potential (MEP) latencies as a function of the distance from the brain. The abductor pollicis brevis muscle (APB), abductor digiti minimi muscle (ADM), flexor carpi radialis muscle (FCR), biceps brachii muscle (BCS), tibialis anterior muscle (TA), and gastrocnemius muscle (GCN) were mapped in the present study

According to the Shapiro–Wilk normality test, MEP latencies were non-normally distributed in both genders. Importantly, the non-normal distribution of the MEP latencies was not adjusted by natural logarithmic transformation. The non-normally distributed MEP latencies for all mapped muscles of both genders are shown in Table 2 and Fig. 1. Furthermore, the differences in MEP latency distributions are compared in Table 2 and Fig. 2 for all mapped muscles. For both genders, median MEP latency values were the highest for GCN and TA, followed by APB and ADM (Table 2; Fig. 2). Accordingly, median MEP latencies derived from FCR and BCS mapping were the lowest when compared to the other mapped muscles (Table 2; Fig. 2).

**Table 2:** Motor evoked potential (MEP) latency by mapped muscle and gender

Gender	Muscle	Min	1st Qu.	Median	3rd Qu.	Max
Male	APB	19.14	22.18	23.29	24.56	30.76
	ADM	18.94	22.00	23.20	24.19	32.50
	FCR	13.66	16.60	18.00	19.22	22.12
	BCS	12.93	14.06	15.69	17.56	20.40
	TA	28.81	31.27	31.83	32.47	34.90
	GCN	28.35	31.11	32.99	35.22	38.27
Female	APB	18.07	20.80	21.63	22.26	32.09
	ADM	17.76	20.44	21.47	22.79	22.79
	FCR	12.75	14.88	16.15	17.19	19.68
	BCS	10.51	12.28	14.15	15.77	15.77
	TA	24.10	28.00	29.68	31.59	34.09
	GCN	28.09	30.04	30.66	32.53	39.50

This table shows the minimum (Min), first quartile (1st Qu.), median, third quartile (3rd Qu.), and maximum (Max) values for motor evoked potential (MEP) latencies by mapped muscle and gender (in ms). In the present study, the abductor pollicis brevis muscle (APB), abductor digiti minimi muscle (ADM), flexor carpi radialis muscle (FCR), biceps brachii muscle (BCS), tibialis anterior muscle (TA), and gastrocnemius muscle (GCN) were mapped

### MEP latency variability

**Fig. 3:**
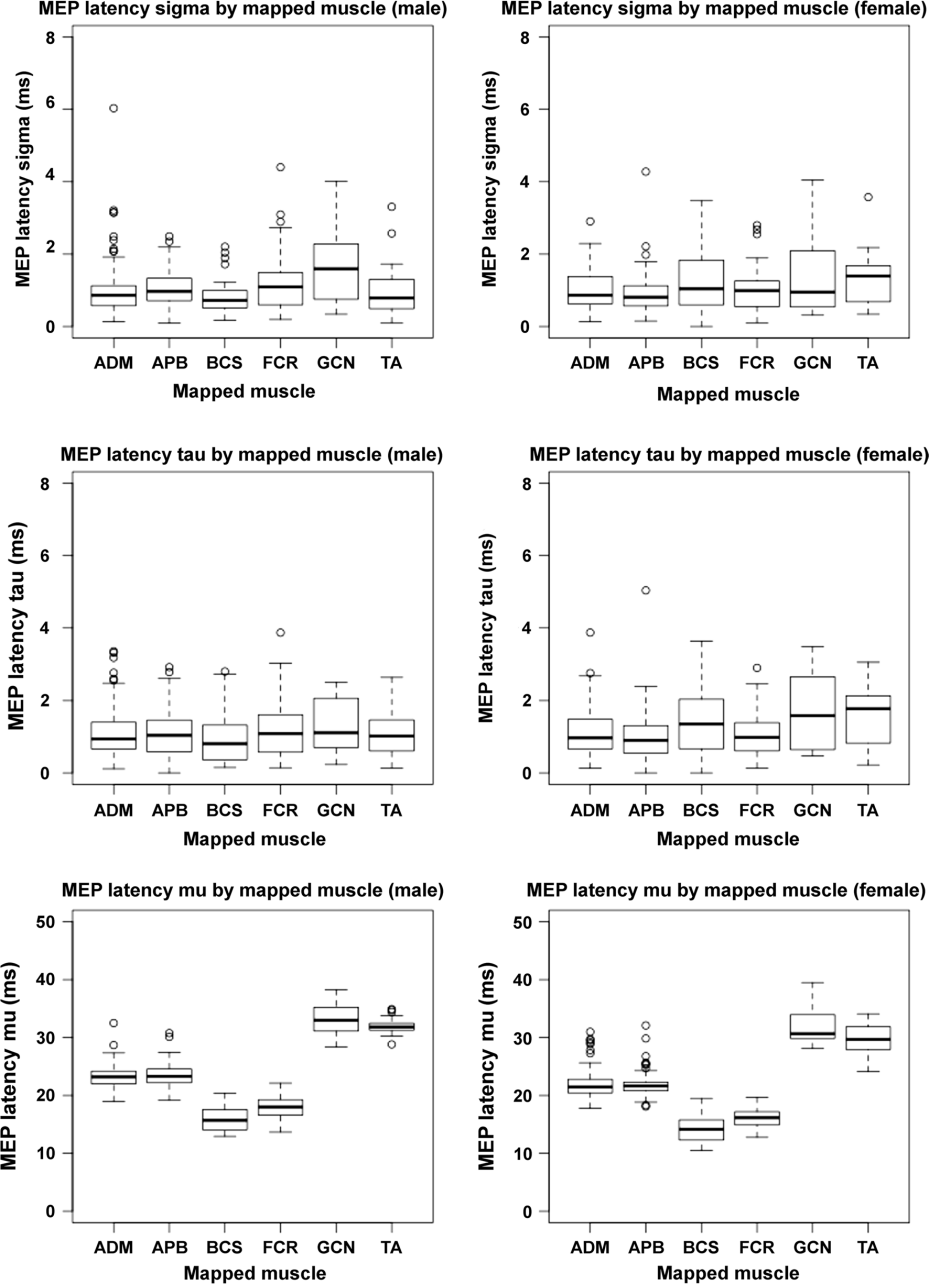
Boxplots showing the distribution of ex-Gaussian measures (mu, sigma, and tau) by mapped gyrus and gender. Again, the results are shown separately for each mapped muscle (*APB* abductor pollicis brevis muscle, *ADM* abductor digiti minimi muscle, *FCR* flexor carpi radialis muscle, *BCS* biceps brachii muscle, *TA* tibialis anterior muscle, *GCN* gastrocnemius muscle), depending on gender

Since MEP latencies showed non-normal distribution, they were adjusted by calculating the ex-Gaussian measures (mu, sigma, and tau) for each mapped muscle and for each patient when the number of MEPs was at least three. The Shapiro–Wilk normality test confirmed that mu was normally distributed for FCR, BCS, TA, and GCN for both genders (Fig. 3). Though normal distribution was not achieved for APB or ADM, outlying scores decreased for these muscles. Thus, linear mixed-effect multiple regression analysis with the forward step-wise model comparison approach was suitable to investigate the factors underlying the variability in MEP latency.

Only motor-positive stimulation spots were taken into account for MEP latency analyses. In total, there were 197 observations from APB-mapped gyri, 192 observations from ADM-mapped gyri, and 157 observations from FCR-mapped gyri. These numbers all exceeded the minimum sample size required for medium effect size (0.15), power (0.80), and α level (0.01) [26–30]. With this constraint, observations from BCS-mapped, TA-mapped, and GCN-mapped gyri were not further assessed to investigate the factors underlying the variability in MEP latencies because they did not have the minimum number of observations required. In this context, MEPs derived from mapping of LE muscles were not available in 66 patients due to missing responses during nTMS with respect to the applied stimulation protocol.

**Fig. 4:**
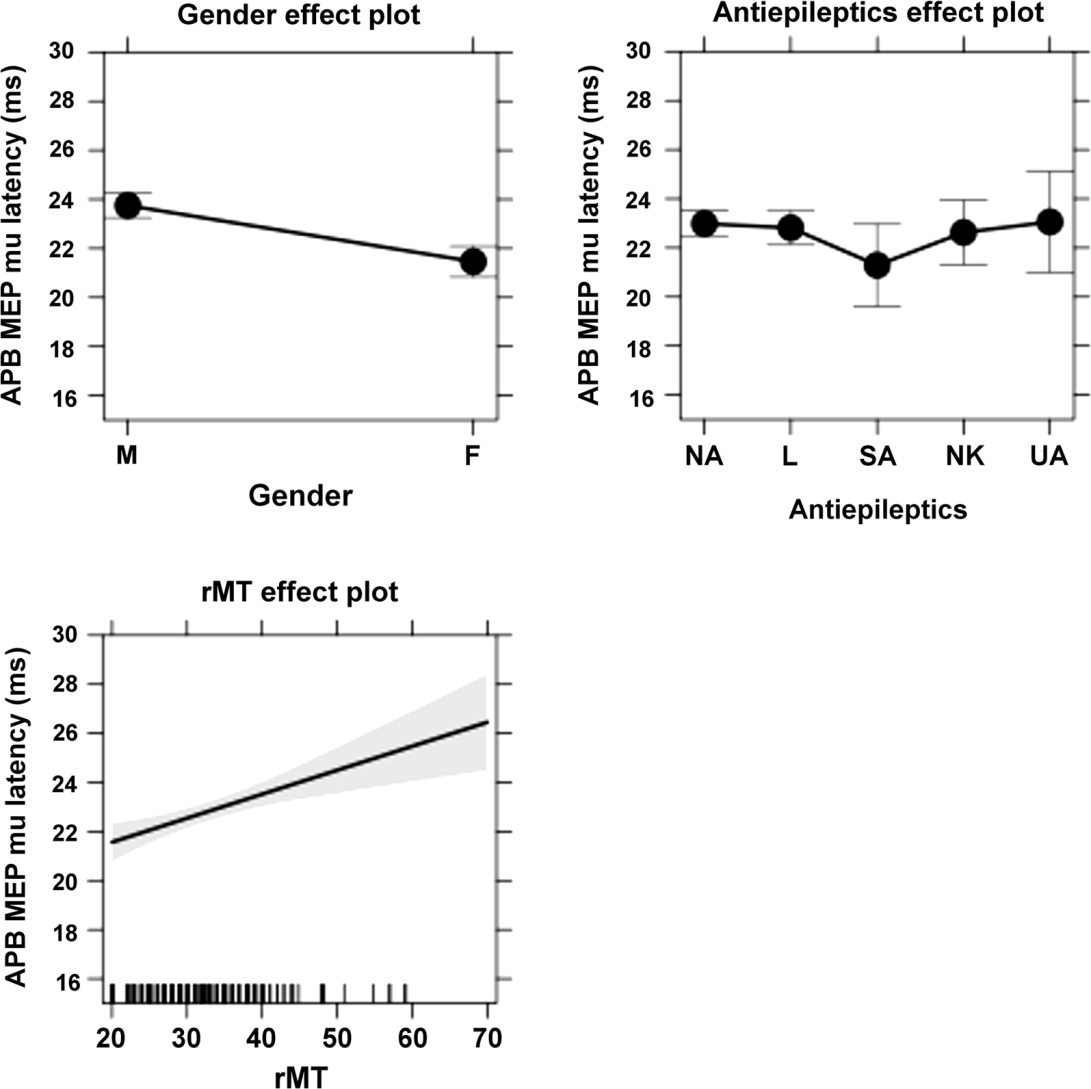
This figure plots the means and confidence intervals (CIs) of the factors that were revealed to be statistically significant regarding motor evoked potential (MEP) latency variability for the abductor pollicis brevis muscle (APB). In this context, gender (*M* male, *F* female), antiepileptics (AEDs: *NA* no AED, *L* levetiracetam, *SA* other specified AEDs, *NK* AED status not known, *UA* unspecified AEDs), and resting motor threshold (rMT, in % of the system’s maximum output) are depicted

**Fig. 5:**
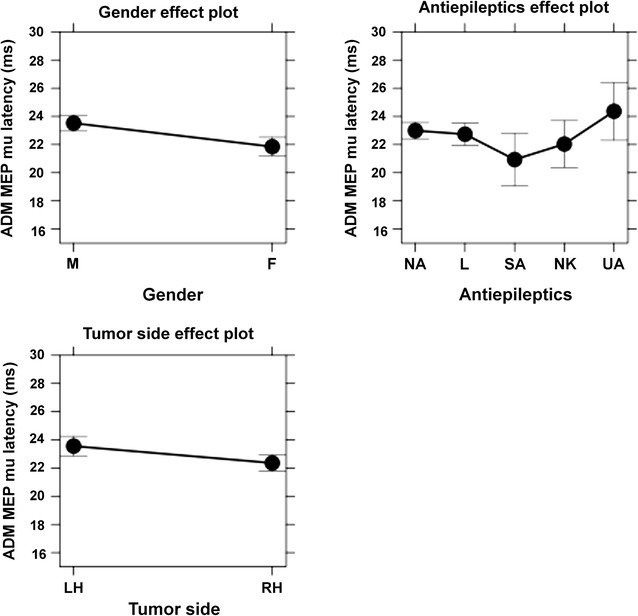
This figure plots the means and confidence intervals (CIs) of the factors that were revealed to be statistically significant regarding motor evoked potential (MEP) latency variability for the abductor digiti minimi muscle (ADM). In this context, gender (*M* male, *F* female), antiepileptics (AEDs: *NA* no AED, *L* levetiracetam, *SA* other specified AEDs, *NK* AED status not known, *UA* unspecified AEDs), and tumor side (*LH* left hemisphere, *RH* right hemisphere) are depicted

**Fig. 6:**
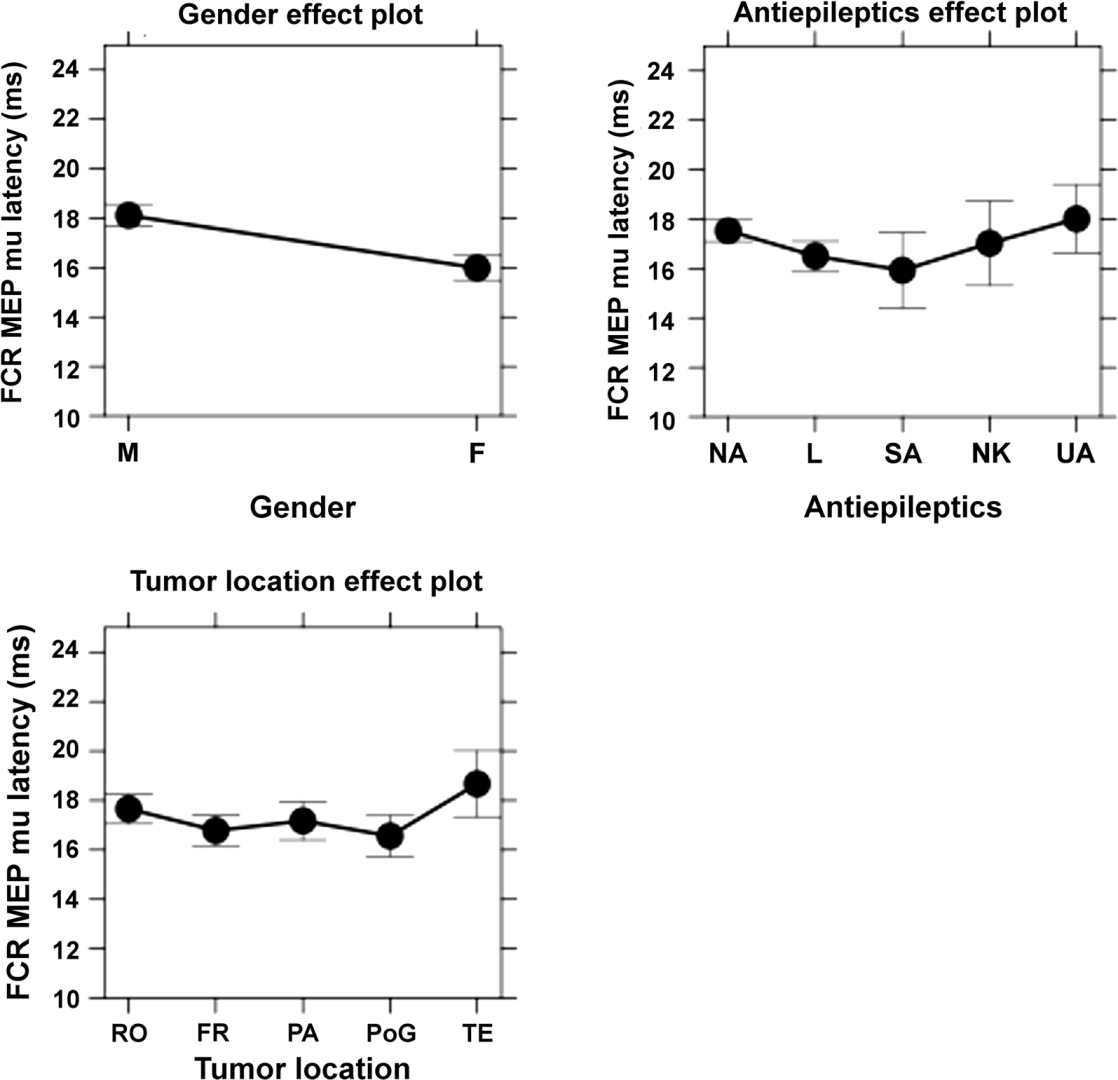
This figure plots the means and confidence intervals (CIs) of the factors that were revealed to be statistically significant regarding motor evoked potential (MEP) latency variability for the flexor carpi radialis muscle (FCR). In this context, gender (*M* male, *F* female), antiepileptics (AEDs: *NA* no AED, *L* levetiracetam, *SA* other specified AEDs, *NK* AED status not known, *UA* unspecified AEDs), and tumor location (*RO* Rolandic, *FR* frontal, *PA* parietal, *PoG* postcentral gyrus, *TE* temporal) are depicted

As a result of the multiple regression analysis, common factors (relevant to APB, ADM, and FCR) and muscle-specific factors (relevant to APB, ADM, or FCR) were identified (Table 3). When the individual differences were partialled out by the random intercept for participants, gender and AED intake were revealed to be common factors underlying MEP latency variability (Table 3). Muscle-specific factors were rMT for APB (mu of the MEP latency was predicted to be longer for patients with higher rMT than for those with lower rMT), tumor side for ADM (mu of the MEP latency was predicted to be longer for patients with left-sided tumors than for those with right-sided tumors), and tumor location for FCR (mu of the MEP latency was predicted to be longer for patients with tumors in the central or temporal regions when compared to patients suffering from tumors within frontal regions or the PoG). The results for significant common and muscle-specific factors underlying the MEP latency variability of the considered muscles are shown in effect plots (Figs. 4, 5, 6).

**Table 3:** Significant predictor variables for motor evoked potential (MEP) latency by mapped muscle

Muscle	Predictors	p
APB MEP latency (mu) (n = 197)	Plus gender	<0.001
	Plus AED	0.012
	Plus rMT	0.008
ADM MEP latency (mu) (n = 192)	Plus gender	0.001
	Plus AED	0.016
	Plus tumor side	0.009
FCR MEP latency (mu) (n = 157)	Plus gender	<0.001
	Plus AED	0.012
	Plus tumor location	0.016

This table shows the predictor variables for MEP latencies that were statistically significant. In this context, common factors (gender and antiepileptics = AED) and muscle-specific factors (resting motor threshold = rMT, tumor side, tumor location) were revealed for abductor pollicis brevis muscle (APB), abductor digiti minimi muscle (ADM), and flexor carpi radialis muscle (FCR)

## Discussion

nTMS-based motor mapping provides multiple neurophysiological variables, out of which mostly MEP amplitude and MEP latency are commonly used to distinguish between motor-positive and motor-negative spots in the clinical setting [4–6]. In numerous studies, MEP latency was shown to remain comparatively stable within individuals, whereas MEP amplitude showed a high intra-individual and inter-individual variability [11–14]. In this context, the present study identified significant factors contributing to MEP latency variability, which were divided into common factors (relevant to APB, ADM, and FCR) and muscle-specific factors (relevant to APB, ADM, or FCR). In the following, we discuss our results on MEP latency variability in patients with brain tumors with regards to the latest literature.

### Common factors underlying MEP latency variability

#### Gender

In our cohort of 100 patients with brain tumors, we identified gender as a common factor underlying MEP mu latency variability in all analyzed muscles (APB, ADM, and FCR). MEP mu latency was predicted to be significantly higher in male than in female patients (Table 3; Figs. 4, 5, 6). However, it is important to mention that body height was not explicitly recorded in the present study, which might confound our findings.

Accordingly, studies on healthy volunteers showed that mean MEP latencies correlate with subjects’ gender, height, and age, respectively [13, 31–33]. After adjusting MEP latencies to height or UE length, no significant differences between men and women were observed. Resulting from that, the gender-related differences in MEP latencies in our study are most likely due to height differences between the genders. Picht et al. reported on similar findings in a cohort of brain tumor patients undergoing preoperative nTMS-based motor mapping: they observed shorter median MEP latencies in the tumorous hemispheres of women than in men [14]. Contrary to Saisanen et al., they did not report any findings of MEP latency influenced by age [13]. In our study, we also could not determine age as an influencing factor, which might be caused by our cohort’s age distribution with mostly middle-aged patients (Table 1).

#### AED intake

In the present study, AED intake was identified as a common factor underlying MEP mu latency variability in all three of the analyzed muscles (APB, ADM, and FCR). During analyses, no AED intake was compared against levetiracetam, other specified AEDs, unspecified AEDs, and unknown AED status (Table 1). In this context, MEP latency was predicted to be slightly higher for unspecified AED intake and lower for specified AEDs. No clear difference was observed for predictions of unknown AED status or levetiracetam, in addition to no AED intake (Table 3; Figs. 4, 5, 6).

In past studies, levetiracetam was described as a potentially beneficial alternative to conventional AEDs in patients with brain tumors, which is based on comparatively rare drug interactions and side effects [34, 35]. Therefore, this drug is increasingly used in the neurosurgical context, allowing formation of a subgroup large enough for statistically meaningful analysis in the present study (Table 1). This was not the case for other AEDs, thus only allowing categorization in other specified or unspecified AEDs. These two groups are inhomogeneous with low numbers of observations, which does not allow attribution of MEP latency variability to a single drug. However, our study raises awareness that AED intake in general has to be considered regarding MEP latencies. More importantly, it is able to show that levetiracetam intake does not predict higher or lower MEP latencies when compared to no AED intake, which has not been investigated previously. This finding indicates that, besides rare drug interactions and side effects, levetiracetam seems to be a favorable drug when it comes to nTMS-based motor mapping because it is not associated with significant alteration in MEP latencies according to our regression analysis.

In this context, the distinct effects of levetiracetam on cortical excitability are not fully understood, which is reflected by a comparatively limited amount of studies that at least partially arrived at opposing results [36–38]. Whereas the excitability in healthy volunteers was not significantly changed after a single oral dose of levetiracetam in one of these studies, the other two approaches described statistically significant rMT increases due to levetiracetam delivery, suggesting a drug-dependent decrease of neuronal excitability [36–38]. Regarding its way of functioning, it is assumed that levetiracetam primarily decreases potassium currents and increases the decay of calcium currents, which is expressed by excitability changes in the context of levetiracetam intake [39, 40]. As revealed by the present study, levetiracetam intake does not seem to predict higher or lower MEP latencies when compared to no AED intake. Hence, changes in excitability might still be present within rMT values or MEP amplitudes, but interestingly, MEP latency does not seem to be significantly affected by this kind of drug intake.

### Muscle-specific factors underlying MEP latency variability

#### rMT for APB

Studies comparing stimulation at threshold level with supra-threshold stimulation of one hemisphere showed that an increase in stimulation intensity leads to a stronger response with increased MEP amplitudes and decreased MEP latencies [23, 32]. On the contrary, we observed that in APB only, higher rMT was associated with an increase of MEP mu latencies. Therefore, our results do not represent changes in MEP latency due to mere stimulation intensity increase, but rather identify the rMT as an APB-specific factor for MEP latency variability (Table 3; Figs. 4, 5, 6).

In healthy volunteers, higher threshold intensities are actually related to longer MEP latencies [19, 32]. Studies assumed that direct corticospinal tracts with faster connections have the lowest thresholds, whereas higher rMT stimulates predominantly indirect or polysynaptic connections and therefore leads to longer MEP latencies. A study on stroke patients presented that in lesioned hemispheres, compared to healthy hemispheres, there are longer MEP latencies and higher rMTs [41]. A study on brain tumor patients also showed that differences in rMT between healthy and impaired hemispheres in tumor patients indeed can be found, although not in all—or even most—patients [14]. In our cohort, we accordingly assume that the higher rMT is an indicator for impairment of motor pathways, which leads to recruitment of indirect connections and therefore a prolonged MEP mu latency. Still, these results have to be considered carefully, as this effect was seen only for one of the three stimulated muscles (APB). Further studies with higher MEP counts for each muscle are necessary to support our current results regarding rMT, and they might reveal the reason why this factor was identified as muscle-specific rather than common.

#### Tumor side for ADM

Interhemispheric differences in neurophysiological parameters are a controversial subject in the literature. As an example, for rMT, there are studies reporting differences between the RH and LH [42], studies reporting differences between DO and NDO hemispheres [43], and studies reporting no interhemispheric difference [18]. For MEP latency, there are many studies on healthy volunteers, which showed no correlation between hemispheres (LH vs. RH nor DO vs. NDO hemispheres), especially not after correction of UE lengths [13, 18, 31, 32]. A study on patients with brain tumors confirmed these findings [14].

Considering our results, we observed that, for ADM only, MEP mu latencies were predicted to be longer in LHs with brain tumors than in RHs with brain tumors according to our multiple regression approach (Table 3; Figs. 4, 5, 6). Although one would assume this is due to the dominancy of the LH, we did not observe this effect in the analysis of DO versus NDO hemispheres. With regards to our patients’ characteristics, gender distribution is not likely to cause this effect either, as both groups are similar (Table 1).

One possible explanation for why the tumor side was an underlying factor of variability in MEP latency while the hemisphere dominancy was not, might be that in left-handed patients we assumed the RH to be the motor-dominant hemisphere. Of course, this is very reductive, as there are different gradations of handedness instead of just-right- or just-left-handedness. Still, this effect might not be of importance, as only 8% of our patients in total were left-handed. In any case, this interpretation remains speculative, and further studies might aim to clarify the finding that tumor side is a relevant factor—a result that has not yet been described in previous investigations. Upcoming studies should also distinctly explore why the tumor side was revealed to be a muscle-specific but not common factor.

#### Tumor location for FCR

In patients with eloquent brain tumors, the lesion frequently impairs the functional connectivity of the brain—either directly due to interaction with the cortical area or the corticospinal tract or indirectly due to a mass effect or edema—and can result in a loss of motor function. The worst deficits are expected when the primary motor cortex is impaired directly by the tumor. This is in accordance with our cohort, as patients with RO tumors tend to suffer from motor deficits most often compared to the other groups (motor deficit: RO 37%, FR 17%, PA 21%, PoG 35%, TE 20%).

We did not identify motor deficit as a significant predictor for MEP mu latency, but for FCR, MEP mu latency in patients with a tumor located in RO or TE regions was predicted to be longer than in those with tumors in PoG or FR areas, whereas MEP mu latencies of PA tumors ranged in the middle (Table 3; Figs. 4, 5, 6). It seems logical that tumors located in RO areas, other than in any other location, induce the largest changes in functional representation as they are situated directly within the primary motor cortex. We are currently aware of only one study researching the variability of MEP latency in brain tumor patients, which did not show any major difference in MEP latency between the healthy and impaired hemisphere [14]. Yet, there is a study on stroke patients showing longer MEP latencies after TMS of affected hemispheres when compared to healthy hemispheres [41]. As for the TE tumor group, these patients tended to have the largest tumor volumes out of all groups, and we assume the longer MEP mu latencies are due to the subcortical spatial relation of tumor tissue to the corticospinal tract and its compression [19].

It is of interest to discern whether these variables are directly associated with motor deficits of contralateral limbs or with mere changes in structural anatomy. The analysis of our cohort indicates the latter, but as these results were significant only for one of three analyzed muscles, further studies with higher MEP counts and different muscles (not solely UE muscles) are crucial.

### Limitations

Despite that this study successfully identified significant common and muscle-specific predictors regarding MEP latency variability in nTMS-based motor mapping, some important shortcomings have to be reported. In this context, we only investigated the lesioned hemisphere, which makes comparisons between diseased and healthy hemispheres within the same patients impossible. However, such interhemispheric comparisons might be useful for assessing the functional status of the motor system with later implications for risk stratification, as demonstrated in a previous study [14]. Although the lack of data analysis among healthy hemispheres in the present study should not restrict the significance of our data per se, such interhemispheric comparison might be considered during upcoming investigations.

Furthermore, our multiple regression analysis solely included a set of UE muscles (APB, ADM, FCR), whereas LE muscles were only initially mapped and recorded by EMG (TA, GCN) but not taken into account during regression analysis. This was due to the fact that the mapping of these LE muscles unfortunately did not lead to numbers of observations needed to run this kind of statistical analysis [26–30], thus restricting our findings to the UE muscles and leaving space for similar approaches in LE muscles, which would require higher MEP counts.

Importantly, body height was not considered as an isolated factor fed into multiple regression analysis. However, since this factor is most likely related to gender, we can certainly assume that it contributed to the identified variability in gender that was revealed as a common predictor variable. In this context, previous studies have repeatedly reported that MEPs recorded from UE muscles are generally correlated to body height [13, 31, 33]. Hence, this factor should be included in future approaches on MEP latency variability among brain tumor patients.

Furthermore, we only used rMTs derived from APB stimulation for mapping of motor areas. This approach allows for differentiation of findings that are specific to single muscles (muscle-specific factors) and findings that are observed in all muscles together (common factors), but only against the background of mapping with a muscle-specific, APB-derived rMT. Thus, our findings are primarily relevant for current mapping procedures that use the APB for rMT determination, which is, however, in line with most previous neurosurgical mapping studies [5, 10, 20–22]. It has to be confirmed whether comparable results are also present for mapping with rMTs derived from other muscles (e.g., ADM or FCR).

### Clinical implications and significance

This is one of the first studies that uses nTMS to systematically explore MEP latency distributions and, in a second step, identifies clinical factors that may underlie MEP latency variability. In this context, there is only a limited amount of literature on MEP characteristics derived from nTMS, which primarily reports results derived from healthy subjects [13, 19]. To the best of our knowledge, there is only one comparable approach among neurosurgical patients available while the use of this technique for neurosurgical mapping strongly increases [14]. Thus, more data on the matter seems to be mandatory for further successful and reliable application of nTMS in neurosurgery. Consequently, the specific need for data on MEP characteristics among neurosurgical patients has already been pointed out in one of the previous investigations in healthy volunteers [19]. Therefore, the results of the present study add knowledge to the limited amount of data on the matter.

While there is a comparatively large body of literature on MEP amplitudes and latency variability and influencing factors derived from stimulation with non-navigated TMS systems [11, 12, 15–17], only a few studies thus far have used nTMS [13, 14, 19]. This situation is of high importance since it is known that only slight variations in coil placement and angulation can lead to different responses [18]. Thus, although previous non-navigated TMS approaches heavily contributed to the knowledge about MEP characteristics, we can assume that non-navigated stimulation might have been confounded by non-optimal coil adjustments, which can limit the validity and reproducibility of such data. In this context, the spatial extent of motor maps, MEP amplitudes at certain mapping points, and MEP latencies might be insufficient due to non-optimal coil placement; a factor that can be controlled during nTMS [3]. While data on the extent of nTMS-based motor maps were not the topic of the present study and have already been published [20], an update on MEP characteristics and influencing factors by the use of nTMS seemed to be necessary in the neurosurgical context.

Regarding influencing factors, at least some of the parameters investigated are specific to brain tumors, which make their assessment impossible in nTMS-based studies among healthy volunteers. In this context, data on tumor-specific factors have solely been investigated in one study so far [14]. Whereas this study predominantly revealed negative results in the sense that no factors except gender were identified as underlying variables of MEP latency variability [14], the present approach revealed more common and muscle-specific factors as significant while considering a larger set of predefined variables.

Nevertheless, the present study only revealed two significant common factors and three muscle-specific factors for MEP latency variability out of a predefined set consisting of 14 clinical factors. This can be regarded as beneficial regarding neurosurgical motor mapping since it demonstrates that only a few variables interfere with MEP latency and, therefore, should be documented and controlled, if possible. However, it is interesting that at least some significant factors have been revealed since MEP latencies have principally shown to be among the most stable parameters during TMS applications, whereas MEP amplitudes, for instance, can considerably vary from stimulus to stimulus due to rather unspecific reasons [11–14].

Despite that nTMS-based mapping in neurosurgery is primarily used for preoperative planning, its application is increasingly expanded to delineation of plastic reshaping in the context of brain tumors. Such plastic changes can occur to different extents, but they have repeatedly been demonstrated by nTMS and intraoperative stimulation [44–47]. Delineation of plastic changes commonly requires follow-up mappings to compare the spatial characteristics of the motor maps. However, some of the significant factors identified in the present study may change during the interval between initial and follow-up mapping (e.g., AED doses), and because they have shown to underlie MEP latency variability, they should be carefully documented and considered to prevent confounding of results when MEP latency is, among others, used as a parameter to distinguish between motor-positive and motor-negative stimulation spots. Such confounding could be due to different mapping hotspots and numbers of motor-positive stimulation points between initial and follow-up mapping, which might be wrongly attributed to plasticity while being rather related to different motor maps due to changed clinical factors. Consequently, the factors identified in the present study should facilitate better control and understanding of nTMS mapping parameters in neurosurgery.

In addition to distinguishing between motor-positive and motor-negative spots, neurophysiological nTMS criteria, such as MEP latency, might be used to assess the functional status of the motor system in the course of a disease like brain tumors, for instance. While first approaches have been made in the context of neurosurgery by interhemispheric comparison of such values [14], they cannot yet be regarded as routine, and more studies are needed to clarify applicability. The present study can be seen as another step that might lead to individual risk stratification based on nTMS characteristics in the long run.

Furthermore, information regarding subcortical tracts can be provided by nTMS when combined with diffusion tensor imaging fiber tracking (DTI FT), as demonstrated in recent studies [48–51]. In this context, MEP latency maps could be used to refine the nTMS-based DTI FT approach in the sense that stimulation spots with the shortest MEP latencies could be used as seed regions to improve motor pathway tracking [51]. In contrast, stimulation points with rather long MEP latencies might be indicative of pathways under stress in the context of tract compressions due to space-occupying brain lesions, as suggested in a recent approach [19]. Again, knowledge about the factors contributing to MEP latency variability is essential for accurate mapping and DTI FT, and it helps to understand underlying neurophysiologic mechanisms. To increasingly exploit these neurophysiological nTMS characteristics in patients with brain tumors that could be supplementary to preoperative motor mapping, further studies are needed.

## Conclusions

Based on a large cohort of neurosurgical patients, this study provides data on MEP latency distributions and a wide range of clinical factors that may underlie MEP latency variability in nTMS-based motor mapping of brain tumor patients. We were able to reveal significant, common factors (gender, associated with height, and AED) as well as muscle-specific factors (rMT, tumor side, tumor location) for MEP latency variability. Whereas the common factors can easily be discussed against the background of previous research (mainly acquired with non-navigated stimulation), some of the findings on significant muscle-specific factors cannot be distinctly clarified by the data of the present or previous studies. Nevertheless, the results of our approach might further refine nTMS-based motor mapping, and they should be taken as a basis for specific research on single, significant muscle-specific factors in the future.

## Abbreviations

II: astrocytoma WHO grade II
III: astrocytoma WHO grade III
IV: astrocytoma WHO grade IV
ADM: abductor digiti minimi muscle
AED: antiepileptic drug
APB: abductor pollicis brevis muscle
BCS: biceps brachii muscle
BMRC: British Medical Research Council
CI: confidence interval
D: deficit
DCS: direct cortical stimulation
DO: dominant
DTI FT: diffusion tensor imaging fiber tracking
E: edema
EMG: electromyography
F: female
FCR: flexor carpi radialis muscle
fMRI: functional magnetic resonance imaging
FR: frontal
GCN: gastrocnemius muscle
L: levetiracetam
LE: lower extremity
LH: left hemisphere
M: male
ME: metastasis
MEG: magnetoencephalography
MEP: motor evoked potential
MRI: magnetic resonance imaging
NA: no AED
ND: no deficit
NDO: non-dominant
NE: no edema
NK: AED status not known
NR: no recurrence
nTMS: navigated transcranial magnetic stimulation
PA: parietal
PoG: postcentral gyrus
R: recurrence
RH: right hemisphere
rMT: resting motor threshold
RO: rolandic
SA: specified AED
SD: standard deviation
TA: tibialis anterior muscle
TE: temporal
TMS: transcranial magnetic stimulation
UA: unspecified AED
UE: upper extremity
X: other entities
Y10: exam year 2010
Y11: exam year 2011
Y12: exam year 2012
Y13: exam year 2013

## References

[1] Picht T (2014). Current and potential utility of transcranial magnetic stimulation in the diagnostics before brain tumor surgery. CNS Oncol.

[2] Ottenhausen M, Krieg SM, Meyer B, Ringel F (2015). Functional preoperative and intraoperative mapping and monitoring: increasing safety and efficacy in glioma surgery. Neurosurg Focus.

[3] Ruohonen J, Karhu J (2010). Navigated transcranial magnetic stimulation. Neurophysiol Clin.

[4] Tarapore PE, Tate MC, Findlay AM, Honma SM, Mizuiri D, Berger MS, Nagarajan SS (2012). Preoperative multimodal motor mapping: a comparison of magnetoencephalography imaging, navigated transcranial magnetic stimulation, and direct cortical stimulation. J Neurosurg.

[5] Krieg SM, Shiban E, Buchmann N, Gempt J, Foerschler A, Meyer B, Ringel F (2012). Utility of presurgical navigated transcranial magnetic brain stimulation for the resection of tumors in eloquent motor areas. J Neurosurg.

[6] Picht T, Schmidt S, Brandt S, Frey D, Hannula H, Neuvonen T, Karhu J, Vajkoczy P, Suess O (2011). Preoperative functional mapping for rolandic brain tumor surgery: comparison of navigated transcranial magnetic stimulation to direct cortical stimulation. Neurosurgery.

[7] Forster MT, Hattingen E, Senft C, Gasser T, Seifert V, Szelenyi A (2011). Navigated transcranial magnetic stimulation and functional magnetic resonance imaging: advanced adjuncts in preoperative planning for central region tumors. Neurosurgery.

[8] Krieg SM, Sabih J, Bulubasova L, Obermueller T, Negwer C, Janssen I, Shiban E, Meyer B, Ringel F (2014). Preoperative motor mapping by navigated transcranial magnetic brain stimulation improves outcome for motor eloquent lesions. Neuro Oncol.

[9] Frey D, Schilt S, Strack V, Zdunczyk A, Rosler J, Niraula B, Vajkoczy P, Picht T (2014). Navigated transcranial magnetic stimulation improves the treatment outcome in patients with brain tumors in motor eloquent locations. Neuro Oncol.

[10] Krieg SM, Sollmann N, Obermueller T, Sabih J, Bulubas L, Negwer C, Moser T, Droese D, Boeckh-Behrens T, Ringel F (2015). Changing the clinical course of glioma patients by preoperative motor mapping with navigated transcranial magnetic brain stimulation. BMC Cancer.

[11] Wassermann EM (2002). Variation in the response to transcranial magnetic brain stimulation in the general population. Clin Neurophysiol.

[12] Kiers L, Cros D, Chiappa KH, Fang J (1993). Variability of motor potentials evoked by transcranial magnetic stimulation. Electroencephalogr Clin Neurophysiol.

[13] Saisanen L, Julkunen P, Niskanen E, Danner N, Hukkanen T, Lohioja T, Nurkkala J, Mervaala E, Karhu J, Kononen M (2008). Motor potentials evoked by navigated transcranial magnetic stimulation in healthy subjects. J Clin Neurophysiol.

[14] Picht T, Strack V, Schulz J, Zdunczyk A, Frey D, Schmidt S, Vajkoczy P (2012). Assessing the functional status of the motor system in brain tumor patients using transcranial magnetic stimulation. Acta Neurochir (Wien).

[15] Darling WG, Wolf SL, Butler AJ (2006). Variability of motor potentials evoked by transcranial magnetic stimulation depends on muscle activation. Exp Brain Res.

[16] Fadiga L, Fogassi L, Pavesi G, Rizzolatti G (1995). Motor facilitation during action observation: a magnetic stimulation study. J Neurophysiol.

[17] Cueva AS, Galhardoni R, Cury RG, Parravano DC, Correa G, Araujo H, Cecilio SB, Raicher I, Toledo D, Silva V (2016). Normative data of cortical excitability measurements obtained by transcranial magnetic stimulation in healthy subjects. Clin Neurophysiol.

[18] Bashir S, Perez JM, Horvath JC, Pascual-Leone A (2013). Differentiation of motor cortical representation of hand muscles by navigated mapping of optimal TMS current directions in healthy subjects. J Clin Neurophysiol.

[19] Kallioniemi E, Pitkanen M, Saisanen L, Julkunen P (2015). Onset latency of motor evoked potentials in motor cortical mapping with neuronavigated transcranial magnetic stimulation. Open Neurol J.

[20] Bulubas L, Sabih J, Wohlschlaeger A, Sollmann N, Hauck T, Ille S, Ringel F, Meyer B, Krieg SM. Motor areas of the frontal cortex in patients with motor eloquent brain lesions. J Neurosurg. 2016;125(6):1431–42. 10.3171/2015.11.JNS152103 26967780

[21] Sollmann N, Tanigawa N, Bulubas L, Sabih J, Zimmer C, Ringel F, Meyer B, Krieg SM. Clinical factors underlying the inter-individual variability of the resting motor threshold in navigated transcranial magnetic stimulation motor mapping. Brain Topogr. 2016. doi:10.1007/s10548-016-0536-9. 10.1007/s10548-016-0536-9 27815647

[22] Krieg SM, Shiban E, Buchmann N, Meyer B, Ringel F (2013). Presurgical navigated transcranial magnetic brain stimulation for recurrent gliomas in motor eloquent areas. Clin Neurophysiol.

[23] Rossini PM, Barker AT, Berardelli A, Caramia MD, Caruso G, Cracco RQ, Dimitrijevic MR, Hallett M, Katayama Y, Lucking CH (1994). Non-invasive electrical and magnetic stimulation of the brain, spinal cord and roots: basic principles and procedures for routine clinical application. Report of an IFCN committee. Electroencephalogr Clin Neurophysiol.

[24] Venables WN, Ripley BD (2002). Modern applied statistics with S.

[25] Fox J (2003). Effects displays in R for generalized linear models. J Stat Soft.

[26] Abramowitz M, Stegun IA (1965). Handbook of mathematical functions.

[27] Cohen J (1988). Statistical power analysis for the behavioral sciences.

[28] Cohen J, Cohen P, West SG, Aiken LS (2003). Applied multiple regression/correlation analysis for the behavioral sciences.

[29] Raudenbush S, Bryk A (2002). Hierarchical linear models.

[30] A priori sample size calculator for hierarchical multiple regression. http://danielsoper.com/statcalc3/calc.aspx?id=16/.

[31] Livingston SC, Goodkin HP, Ingersoll CD (2010). The influence of gender, hand dominance, and upper extremity length on motor evoked potentials. J Clin Monit Comput.

[32] van der Kamp W, Zwinderman AH, Ferrari MD, van Dijk JG (1996). Cortical excitability and response variability of transcranial magnetic stimulation. J Clin Neurophysiol.

[33] Livingston SC, Friedlander DL, Gibson BC, Melvin JR (2013). Motor evoked potential response latencies demonstrate moderate correlations with height and limb length in healthy young adults. Neurodiagn J.

[34] Iuchi T, Kuwabara K, Matsumoto M, Kawasaki K, Hasegawa Y, Sakaida T (2015). Levetiracetam versus phenytoin for seizure prophylaxis during and early after craniotomy for brain tumours: a phase II prospective, randomised study. J Neurol Neurosurg Psychiatry.

[35] Fonkem E, Bricker P, Mungall D, Aceves J, Ebwe E, Tang W, Kirmani B (2013). The role of levetiracetam in treatment of seizures in brain tumor patients. Front Neurol.

[36] Reis J, Wentrup A, Hamer HM, Mueller HH, Knake S, Tergau F, Oertel WH, Rosenow F (2004). Levetiracetam influences human motor cortex excitability mainly by modulation of ion channel function—a TMS study. Epilepsy Res.

[37] Sohn YH, Kaelin-Lang A, Jung HY, Hallett M (2001). Effect of levetiracetam on human corticospinal excitability. Neurology.

[38] Solinas C, Lee YC, Reutens DC (2008). Effect of levetiracetam on cortical excitability: a transcranial magnetic stimulation study. Eur J Neurol.

[39] Madeja M, Margineanu DG, Gorji A, Siep E, Boerrigter P, Klitgaard H, Speckmann EJ (2003). Reduction of voltage-operated potassium currents by levetiracetam: a novel antiepileptic mechanism of action?. Neuropharmacology.

[40] Niespodziany I, Klitgaard H, Margineanu DG (2001). Levetiracetam inhibits the high-voltage-activated Ca(2+) current in pyramidal neurones of rat hippocampal slices. Neurosci Lett.

[41] Cakar E, Akyuz G, Durmus O, Bayman L, Yagci I, Karadag-Saygi E, Gunduz OH. The relationships of motor-evoked potentials to hand dexterity, motor function, and spasticity in chronic stroke patients: a transcranial magnetic stimulation study. Acta Neurol Belg. 2016;116(4):481–7. 10.1007/s13760-016-0633-2 27037821

[42] Koski L, Schrader LM, Wu AD, Stern JM (2005). Normative data on changes in transcranial magnetic stimulation measures over a ten hour period. Clin Neurophysiol.

[43] Triggs WJ, Calvanio R, Macdonell RA, Cros D, Chiappa KH (1994). Physiological motor asymmetry in human handedness: evidence from transcranial magnetic stimulation. Brain Res.

[44] Forster MT, Senft C, Hattingen E, Lorei M, Seifert V, Szelenyi A (2012). Motor cortex evaluation by nTMS after surgery of central region tumors: a feasibility study. Acta Neurochir (Wien).

[45] Robles SG, Gatignol P, Lehericy S, Duffau H (2008). Long-term brain plasticity allowing a multistage surgical approach to World Health Organization Grade II gliomas in eloquent areas. J Neurosurg.

[46] Southwell DG, Hervey-Jumper SL, Perry DW, Berger MS. Intraoperative mapping during repeat awake craniotomy reveals the functional plasticity of adult cortex. J Neurosurg. 2016;124(5):1460–69. 10.3171/2015.5.JNS142833 26544767

[47] Takahashi S, Jussen D, Vajkoczy P, Picht T (2012). Plastic relocation of motor cortex in a patient with LGG (low grade glioma) confirmed by NBS (navigated brain stimulation). Acta Neurochir (Wien).

[48] Krieg SM, Buchmann NH, Gempt J, Shiban E, Meyer B, Ringel F (2012). Diffusion tensor imaging fiber tracking using navigated brain stimulation—a feasibility study. Acta Neurochir (Wien).

[49] Conti A, Raffa G, Granata F, Rizzo V, Germano A, Tomasello F (2014). Navigated transcranial magnetic stimulation for “somatotopic” tractography of the corticospinal tract. Neurosurgery.

[50] Frey D, Strack V, Wiener E, Jussen D, Vajkoczy P, Picht T (2012). A new approach for corticospinal tract reconstruction based on navigated transcranial stimulation and standardized fractional anisotropy values. NeuroImage.

[51] Weiss C, Tursunova I, Neuschmelting V, Lockau H, Nettekoven C, Oros-Peusquens AM, Stoffels G, Rehme AK, Faymonville AM, Shah NJ (2015). Improved nTMS- and DTI-derived CST tractography through anatomical ROI seeding on anterior pontine level compared to internal capsule. Neuroimage Clin.

